# Comparison of characteristics of culture-negative pyogenic spondylitis and tuberculous spondylitis: a retrospective study

**DOI:** 10.1186/s12879-016-1897-0

**Published:** 2016-10-12

**Authors:** Chung-Jong Kim, Eun Jung Kim, Kyoung-Ho Song, Pyoeng Gyun Choe, Wan Beom Park, Ji Hwan Bang, Eu Suk Kim, Sang Won Park, Hong-Bin Kim, Myoung-don Oh, Nam Joong Kim

**Affiliations:** 1 Department of Internal Medicine, Seoul National University College of Medicine, 101 Daehak-ro Jongno-gu, Seoul, 110-744 Republic of Korea; 2 Department of Internal Medicine, Seoul National University Bundang Hospital, Seongnam-si, Gyeonggi-do Republic of Korea; 3 Department of Internal Medicine, Ewha Womans University Mokdong Hospital, Seoul, Republic of Korea

**Keywords:** Spondylitis, Discitis, Epidural abscess, *Mycobacterium tuberculosis*, Spinal tuberculosis

## Abstract

**Background:**

Differences between the characteristics of culture positive pyogenic spondylitis (CPPS) and tuberculous spondylitis (TS) are well known. However, differences between the characteristics of culture negative pyogenic spondylitis (CNPS) and TS have not been reported; these would be more helpful in clinical practice especially when initial microbiologic examination of blood and/or biopsy tissue did not reveal the causative bacteria in patients with infectious spondylitis.

**Methods:**

We performed a retrospective review of the medical records of patients with CNPS and TS. We compared the characteristics of 71 patients with CNPS with those of 94 patients with TS.

**Results:**

Patients with TS had more previous histories of tuberculosis (9.9 vs 22.3 %, *p* = 0.034), simultaneous tuberculosis other than of the spine (0 vs 47.9 %, *p* < 0.001), and positive results in the interferon-gamma release assay (27.6 vs 79.2 %, *p* < 0.001). Fever (15.5 vs. 31.8 %, *p* = 0.018), psoas abscesses (15.5 vs 33.0 %, *p* = 0.011), and paravertebral abscesses (49.3 vs. 74.5 %, *p* = 0.011) were also more prevalent in TS than CNPS.

**Conclusions:**

Different from or contrary to the previous comparisons between CPPS and TS, fever, psoas abscesses, and paravertebral abscesses are more common in patients with TS than in those with CNPS.

## Background

Infectious spondylitis is an infectious disease of the spine or paraspinal structures that can be caused by various microorganisms. It is divided into three categories according to the causative microorganism, namely pyogenic, tuberculous, and brucellar [1]. Brucellar spondylitis is less prevalent in most countries, and pyogenic and tuberculous spondylitis are the main causes of spinal infections. The incidence of infectious spondylitis has been reported to be 2.4 cases per 100,000 persons, with the rate increasing with age [2]. Although the rates of pyogenic spondylitis and tuberculous spondylitis vary regionally, the incidence of pyogenic spondylitis is increasing.

When we manage patients with infectious spondylitis, we obtain tissue cultures and/or blood cultures to identify the causative organism. In pyogenic spondylitis (PS), culture positive rates of tissues and/or blood are between 30 and 70 %, and more than a third of patients are regarded as having culture-negative pyogenic spondylitis (CNPS) [3–6]. In tuberculous spondylitis (TS), culture positive rates of tissues are between 50 and 70 % [7, 8], but culture results are only reported more than 3 weeks after obtaining tissue for microbiologic diagnosis. It is difficult to select the initial empirical treatment regimen when bacterial culture does not reveal the causative microorganism. In such circumstances we might be helped by previous reports comparing the characteristics of PS and TS [1, 9–13]. However the available reports compare the characteristics of culture-positive pyogenic spondylitis (CPPS) with TS, and they may be misleading because the characteristics of CNPS differ from those of CPPS. Information about the differences between CNPS and TS would be much more helpful. In this study we compare the characteristics of CNPS and TS, with the aim of identifying factors which would aid differential diagnosis in the initial clinical setting.

## Methods

### Study population

We retrospectively searched records for patients diagnosed with tuberculous or culture negative pyogenic spondylitis in Seoul National University Bundang hospital in South Korea from March 2004 through December 2014. The study hospital is a 900-bed university-affiliated tertiary hospital. We included cases of infectious spondylitis that met the definition below. Exclusion criteria were: 1) pediatric patients under 15 years of age, 2) culture of spinal tissue and/or blood grew fungi or bacteria other than *Mycobacterium tuberculosis*, 3) no spinal tissue was obtained for microorganism culture.

### Definitions

Infectious spondylitis was defined using clinical and radiological criteria, as in our previous report [4]. The clinical criteria included the presence of compatible symptoms or signs of spondylitis, such as pain in the vertebral area, fever, and neurological symptoms or signs in the vertebrae or extremities. The radiological criteria included findings compatible with spondylitis on magnetic resonance imaging (MRI) [1, 14].

Tuberculous spondylitis was diagnosed when one or more of the following criteria was met:

1. Growth of *M. tuberculosis* in spinal tissue culture
2. Positive *M. tuberculosis* polymerase chain reaction (PCR) test with pathological findings compatible with granulomatous inflammation with necrosis
3. Compatible pathological findings in a spinal tissue biopsy, with cultures negative for ordinary bacteria, and the patient improving in response to anti-tuberculous chemotherapy
4. MRI findings compatible with infectious spondylitis, and chest computed tomographic findings compatible with miliary or active pulmonary tuberculosis.

Culture-negative pyogenic spondylitis was diagnosed when *all* of the following criteria were met:

1. Bacterial culture of spinal tissue grew no microorganisms. If blood cultures were performed, they also grew no bacteria.
2. The symptoms and signs of infectious spondylitis improved with empirical antibiotics ineffective against *M. tuberculosis*
3. *M. tuberculosis* PCR was negative if performed.

### Data variables

The variables collected included baseline characteristics (age, sex, and underlying diseases), presenting symptoms, laboratory data (white blood cell (WBC) count and C-reactive protein (CRP), interferon gamma release assay (IGRA)), and radiological data (involved vertebral area, number of involved vertebral bodies, and presence and location of abscesses). The IGRA was performed using the T-SPOT.TB (Oxford Immunotec, Abingdon, UK) assay according to the manufacturer’s instructions. *M. tuberculosis* PCR using spinal tissue was performed by real-time PCR targeting the senX3-regX3 intergenic region, as described previously [15].

### Statistical analysis

Statistical analysis was performed using SPSS ver. 20.0. The chi-square test and Student’s *t*-test were used to compare baseline characteristics and laboratory and radiological data.

### Study approval

This study was approved by the Institutional Review Board of Seoul National University Bundang Hospital.

## Results

### Baseline characteristics

During the study period, 94 patients with TS and 71 with CNPS were identified. Of the 94 patients with TS, 30 had concomitant pulmonary tuberculosis including 9 with miliary tuberculosis. TS was diagnosed based on positive AFB culture (*n* = 58, 61.7 %), positive *M. tuberculosis* PCR with compatible pathology (*n* = 16, 17.0 %), compatible pathology with response to anti-tuberculous treatment (*n* = 13, 13.8 %), and imaging findings compatible with miliary or active pulmonary tuberculosis (*n* = 7, 7.4 %). The median time to positivity of spine tissue AFB culture was 21 (interquartile range (IQR) 17–27) days in liquid medium and 29 (IQR 24–34) days on solid medium.

The baseline demographic characteristics of the patients are summarized in Table 1. Patients with CNPS were older than those with TS, and had more frequently undergone epidural procedures. The patients with TS more often had concurrent pulmonary and/or extra-pulmonary tuberculosis including miliary tuberculosis (*n* = 9), renal tuberculosis (*n* = 3), tuberculous lymphadenopathy (*n* = 2), and tuberculous pleurisy (*n* = 1). Seventeen patients (23.9 %) had taken antibiotics before being diagnosed with CNPS. The IGRA was performed in 76 patients: 48 with TS and 29 with CNPS. The result of the IGRA was indeterminate in two CNPS patients and one TS patient. One patient in the CNPS group had previously had a positive IGRA performed prior to the development of infectious spondylitis. The proportion of patients with a positive IGRA was significantly higher in the TS group (79.2 %), although 27.6 % of the CNPS were IGRA positive (Table 2).

**Table 1:** Demographic characteristics of patients with tuberculous spondylitis and culture negative pyogenic spondylitis

	CNPS (*N* = 71)	TS (*N* = 94)	*P*-value
Age, mean ± S.D (years)	65.9 (±14.4)	59.2 (±20.0)	0.017
Sex (male, %)	32 (45.1 %)	37 (39.4 %)	0.462
Underlying disease			
Hypertension	34 (47.9 %)	24 (25.5 %)	0.003
Diabetes mellitus	17 (23.9 %)	16 (17.0 %)	0.271
Chronic kidney disease	4 (5.6 %)	3 (3.2 %)	0.465^‡^
Congestive heart failure	0	2 (2.1 %)	0.507^‡^
Liver cirrhosis	6 (8.5 %)	1 (1.1 %)	0.043^‡^
Solid tumor	7 (9.9 %)	2 (2.1 %)	0.040^‡^
Hematologic malignancy	1 (1.4 %)	0	0.430^‡^
Concomitant corticosteroid use	2 (2.8 %)	1 (1.1 %)	0.578
Epidural procedure within 1 year	19 (26.8 %)	9 (9.6 %)	0.004
Foreign body in affected vertebrae ^a^	8 (11.3 %)	3 (3.2 %)	0.057^‡^
Vertebroplasty cement in affected vertebrae	7 (9.9 %)	13 (13.8 %)	0.439
Previous bacteremia within 3 months	3 (4.2 %)	0	0.078^‡^
History of tuberculosis	7 (9.9 %)	21 (22.3 %)	0.034
Concurrent pulmonary tuberculosis	0	30 (31.9 %)	<0.001
Concurrent extrapulmonary tuberculosis^†^	0	15 (16.0 %)	<0.001

*Abbreviations: S.D* standard deviation, *TS* tuberculous spondylitis, *CNPS* culture negative pyogenic spondylitis

^a^Foreign body included Screw or spinal plate for spine fixation

^†^Tuberculous spondylitis was excluded in concurrent extrapulmonary tuberculosis

^‡ †^Fisher’s exact test

**Table 2:** Clinical and laboratory characteristics of patients with tuberculous spondylitis and culture negative pyogenic spondylitis

	CNPS (*N* = 71)	TS (*N* = 94)	*P*-value
Duration of symptoms			
<1 month	32 (45.1 %)	25 (26.6 %)	0.002
1–3 months	32 (45.1 %)	44 (46.8 %)	
> 4 months	7 (9.9 %)	25 (26.6 %)	
Neurologic stage			
No pain	0	3 (3.2 %)	0.164
Spine pain	28 (39.4 %)	52 (55.3 %)	
Radiating pain	38 (53.5 %)	26 (26.6 %)	
Weakness	5 (7.0 %)	13 (13.8 %)	
Sepsis	7 (9.9 %)	15 (16.0 %)	0.254
Initial body temperature over 38 °C	11 (15.5 %)	27 (31.8 %)	0.018
WBC (/mm^3^) (median with IQR)	8600 (7070–11,100)	7890 (6098–10,293)	0.030
CRP ^a^ (mg/dL) (median with IQR)	4.3 (2.4–8.1)	2.8 (1.0–7.2)	0.018
IGRA ^b^			
Positive	8 (27.6 %)	38 (79.2 %)	<0.001
Negative	19 (65.5 %)	9 (18.8 %)	
Psoas abscess	11 (15.5 %)	31 (33.0 %)	0.011
Paravertebral abscess	35 (49.3 %)	70 (74.5 %)	0.001
Epidural abscess	39 (54.9 %)	50 (53.2 %)	0.824
Involved vertebrae			
Cervical	2 (2.8 %)	3 (3.2 %)	N.A.
Cervicothoracic	0	6 (6.4 %)	
Thoracic	5 (7.0 %)	25 (26.6 %)	
Thoracolumbar	3 (4.2 %)	9 (9.6 %)	
Lumbar	47 (66.2 %)	43 (45.7 %)	
Lumbosacral	14 (19.7 %)	6 (6.4 %)	
Sacral	0	1 (1.1 %)	
Cervical and Lumbar (skipped lesion)	0	1 (1.1 %)	
Number of vertebrae involved			
1–2	58 (81.7 %)	65 (69.1 %)	0.067
3–5	13 (18.3 %)	29 (30.9 %)	

*Abbreviations: WBC* white blood cell count, *IQR* interquartile range, *CRP* C-reactive protein, *IGRA* Interferon gamma release assay, *TS* tuberculous spondylitis, *CNPS* culture negative pyogenic spondylitis, *N.A* not applicable

^a^C-Reactive Protein value was missing in 1 case of tuberculous spondylitis

^b^The IGRA was performed in 76 patients: 48 with TS and 29 with CNPS. IGRA result was indeterminate in one case of tuberculous spondylitis and two cases of culture negative pyogenic spondylitis

### Clinical and radiographic characteristics of infectious spondylitis

The clinical, laboratory, and radiologic findings of the patients are shown in Table 2. Duration of symptoms was shorter in patients with CNPS. Patients with CNPS had radiating pain more frequently than those with TS, while weakness was more frequent in TS. Fever was uncommon in CNPS (15.5 %), while one third of the patients with TS had fever at initial presentation. Median CRP level was higher in patients with CNPS (4.3 vs 2.8 mg/dL, *p* = 0.018). In a sub-analysis of the patients with TS, median CRP level was higher when accompanied by pulmonary or miliary tuberculosis (4.2 vs. 1.8 mg/dL, *p* = 0.017).

In both groups, the lumbar spine was the site most frequently affected, with lumbar involvement in 90.1 % of the CNPS patients and 62.8 % of the TS patients. The thoracic spine was involved more frequently in the TS group than the CNPS group (11.3 vs. 42.6 %, *p* < 0.01). Psoas and paravertebral abscesses were significantly more prevalent in patients with TS, whereas epidural abscesses were equally common in the two groups.

## Discussion

In this study we compared the clinical characteristics of CNPS and TS patients at their initial hospital visits. Age, history of epidural procedure within 1 year, concurrent extra-spinal tuberculosis, duration of symptoms, presence of fever, results of IGRA, WBC counts, CRP level, vertebral level involved, and presence of psoas or paravertebral abscess differed significantly between the two groups, and might be useful for differentiating them.

Patients with pyogenic spondylitis have a variety of different clinical presentations. Some have concomitant bacteremia and are septic, with higher levels of acute-phase reactant [16]. By contrast, others have mild disease with lower levels of acute-phase reactant [4]. Several reports have compared the characteristics of patients with CNPS and CPPS. Fever and paravertebral abscesses were less common and erythrocyte sedimentation rate (ESR) and CRP levels were lower in CNPS than in CPPS, which could be explained by the lower bacterial burden in CNPS [5, 17, 18]. Sometimes we are obliged to choose between antibiotics and anti-tuberculous medication for patients with infectious spondylitis, especially when bacterial culture reveals no etiologic organism. At such times we might be guided by reports comparing the characteristics of PS and TS, but this might be misleading because most such reports described differences between CPPS and TS rather than between CNPS and TS. Most of the studies comparing CPPS and TS reported that fever was more common and acute-phase reactants, such as CRP, WBC count, and ESR, were higher in PS than TS, while a more indolent clinical course, concurrent active tuberculosis of other organs, and involvement of thoracic spines were suggestive of TS [1, 11–13, 19].

Many of our findings are in agreement with the above differences between CPPS and TS. However, several differ. First, fever was related to TS not CNPS. Second, paravertebral or psoas abscesses were also more frequent in TS than CNPS. Although not performed in all patients, the IGRA also had diagnostic value in differentiating TS from CNPS. Several studies revealed that fever was more frequent in CPPS than TS, and paravertebral or psoas abscesses were equally common in the two [1, 11, 13, 19]. In our opinion, the lower bacterial burden in patients with CNPS might be associated with the lower rates of fever and paravertebral or psoas abscesses in CNPS than in CPPS or even TS.

Our study had several limitations. First the definition of CNPS was strict and some patients with CNPS may have been excluded; since we included patients who responded to empirical antibiotic regimens, severe cases may have been excluded if their treatment failed. Second, the results of the IGRA should be interpreted with care because the prevalence of tuberculosis and positive rates of IGRA differ between countries.

## Conclusions

In conclusion, CNPS and TS can be differentiated on the basis of several characteristics: older age, higher CRP, previous epidural procedures are suggestive of CNPS. Concurrent active tuberculosis other than in the spine, more indolent course, and involvement of the thoracic spine are suggestive of TS. The presence of fever, psoas abscesses, and paravertebral abscesses are also suggestive of TS.

## Abbreviations

AFB: Acid fast bacilli
CNPS: Culture-negative pyogenic spondylitis
CPPS: Culture-positive pyogenic spondylitis
CRP: C-reactive protein
ESR: Erythrocyte sedimentation rate
IGRA: Interferon gamma release assay
IQR: Interquartile range
MRI: Magnetic resonance imaging
PCR: Polymerase chain reaction
PS: Pyogenic spondylitis
TS: Tuberculous spondylitis
WBC: White blood cell count
